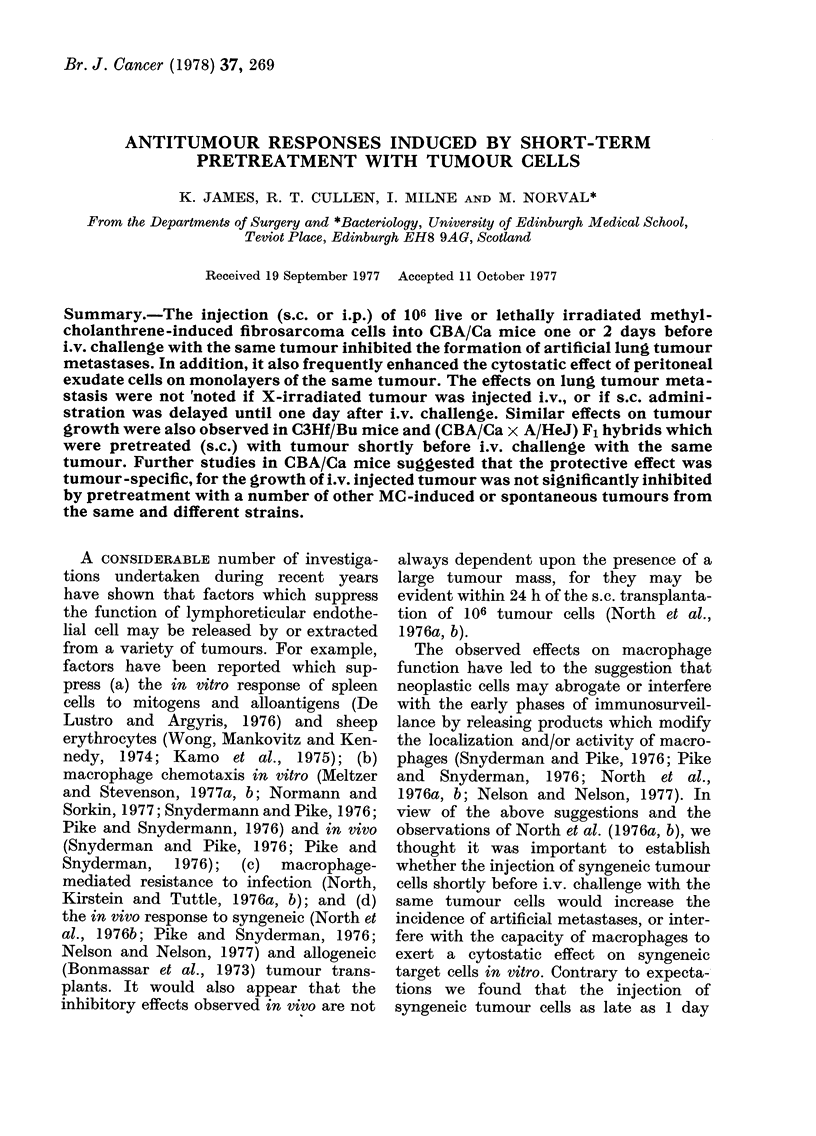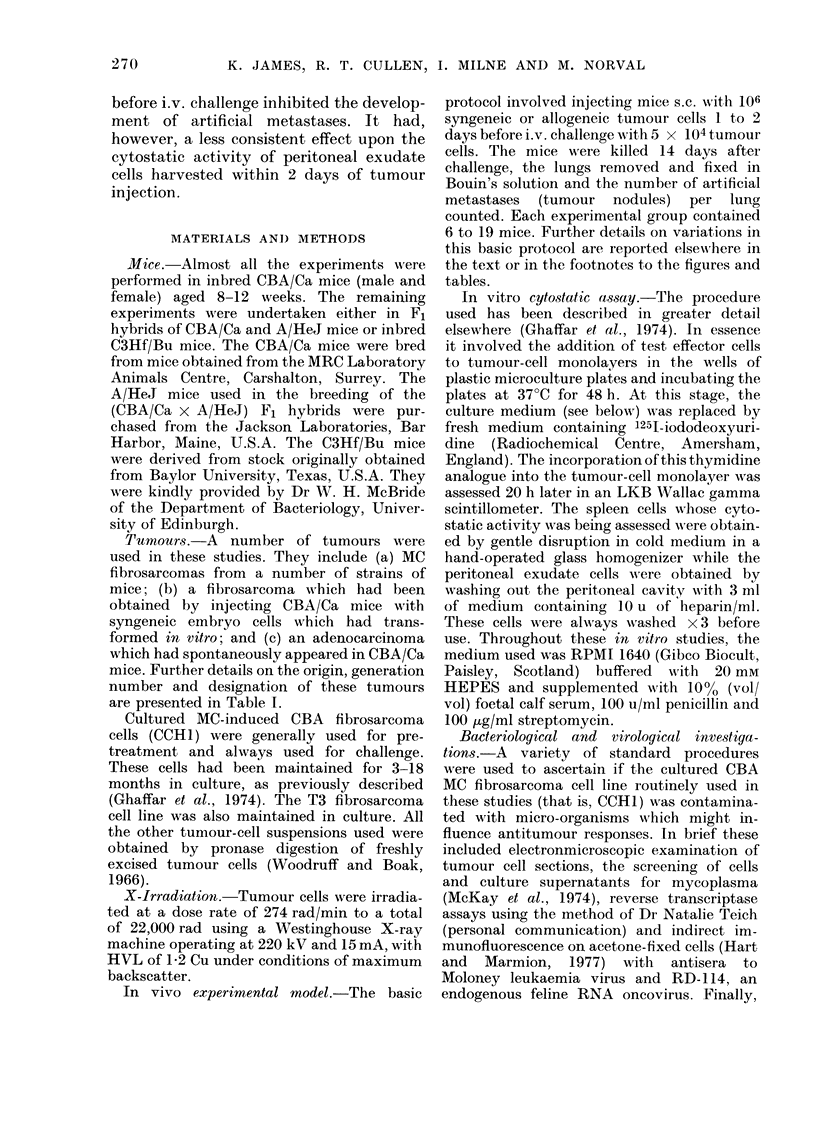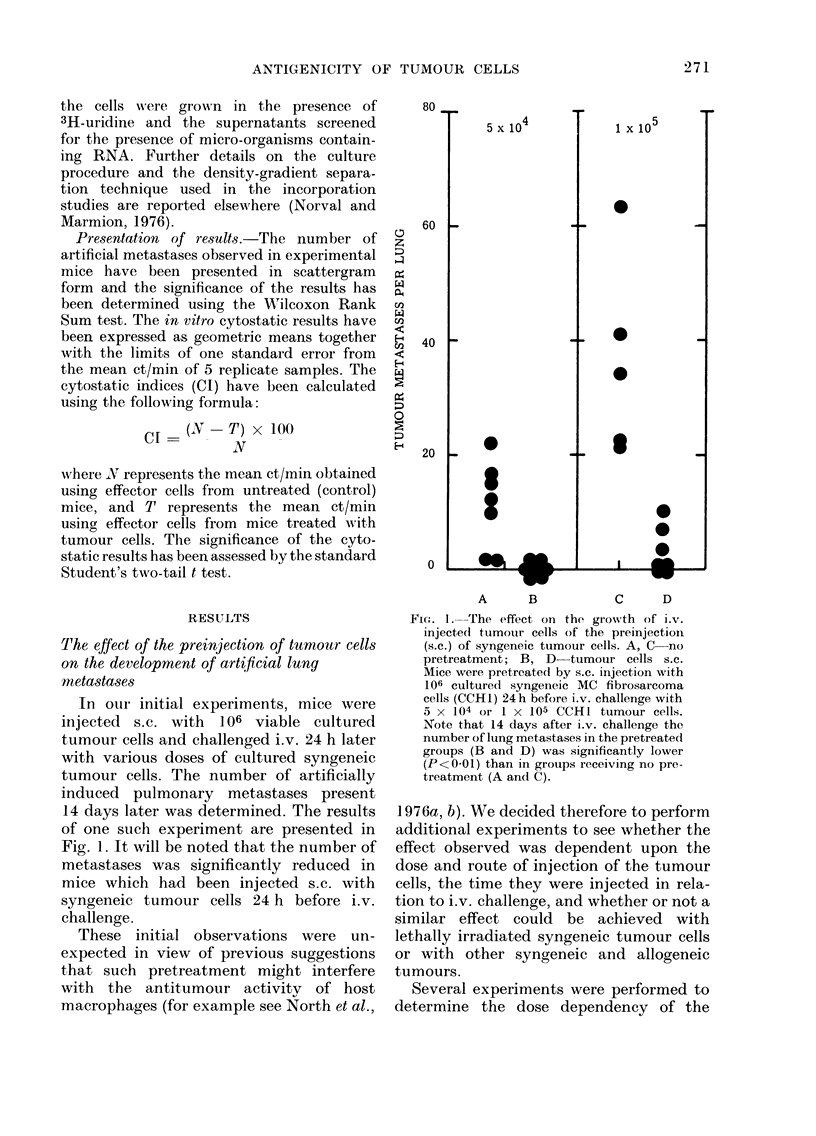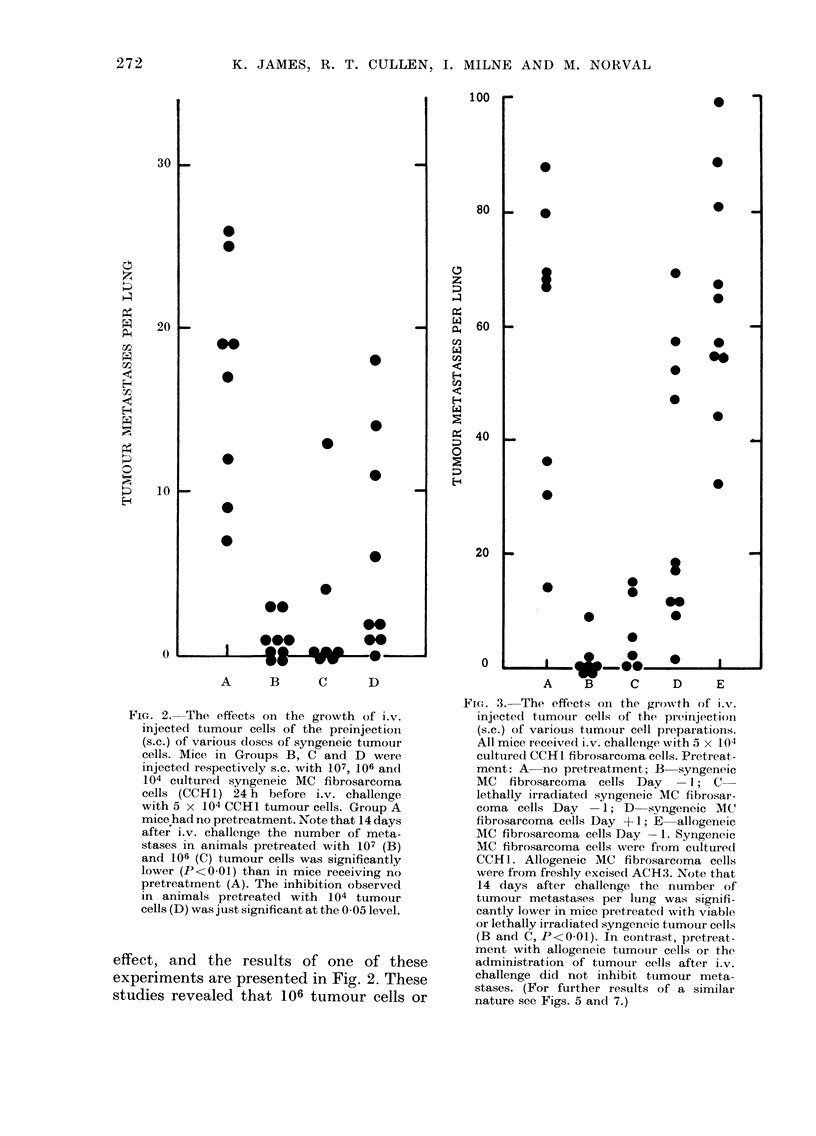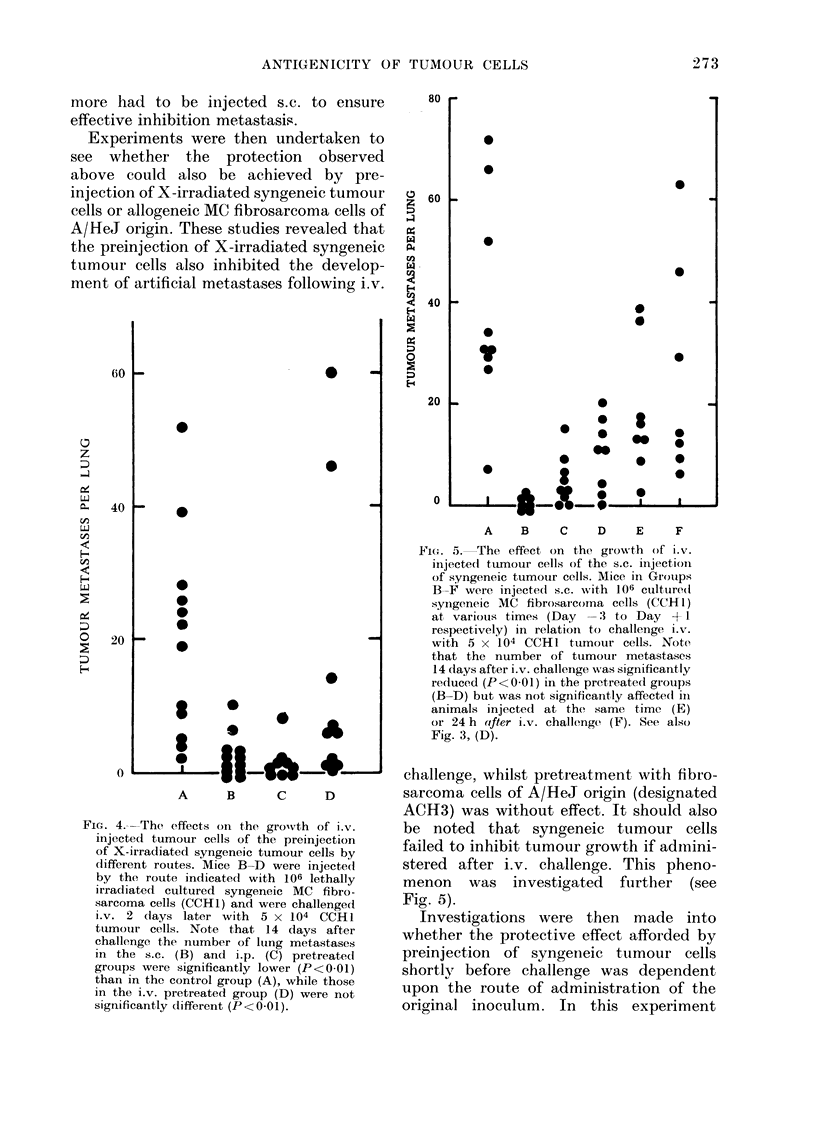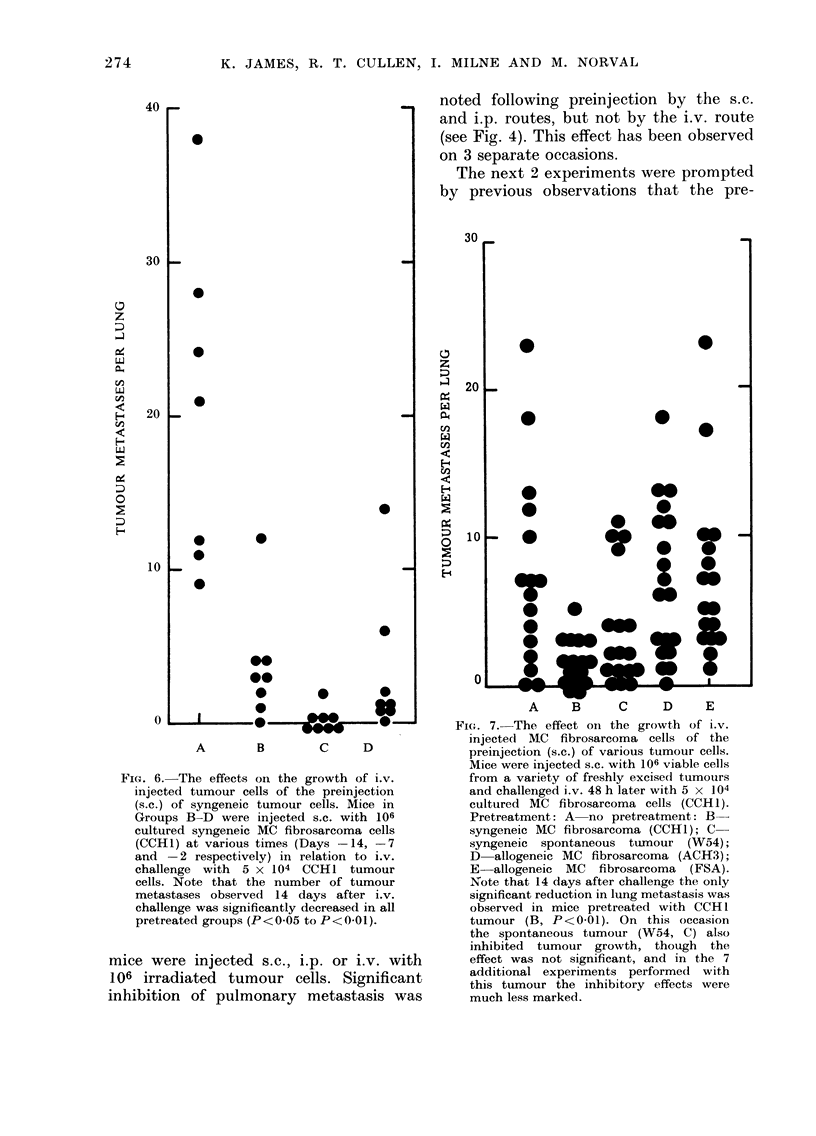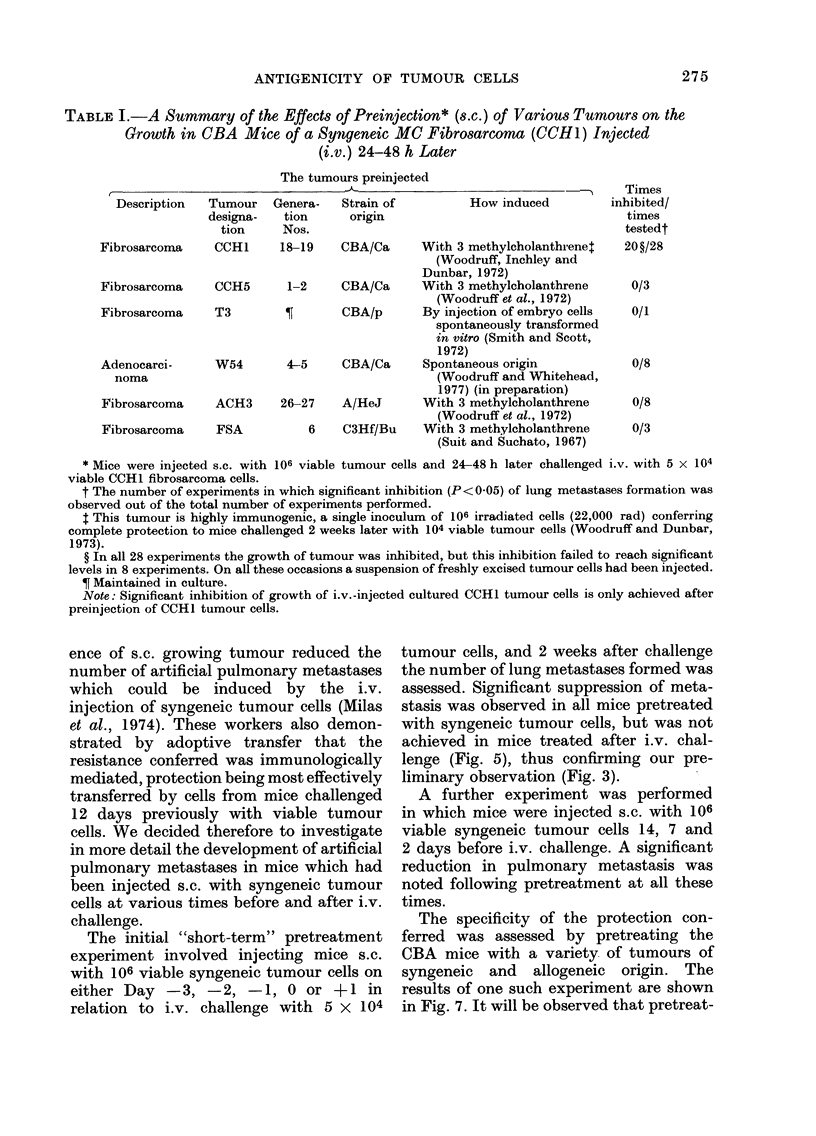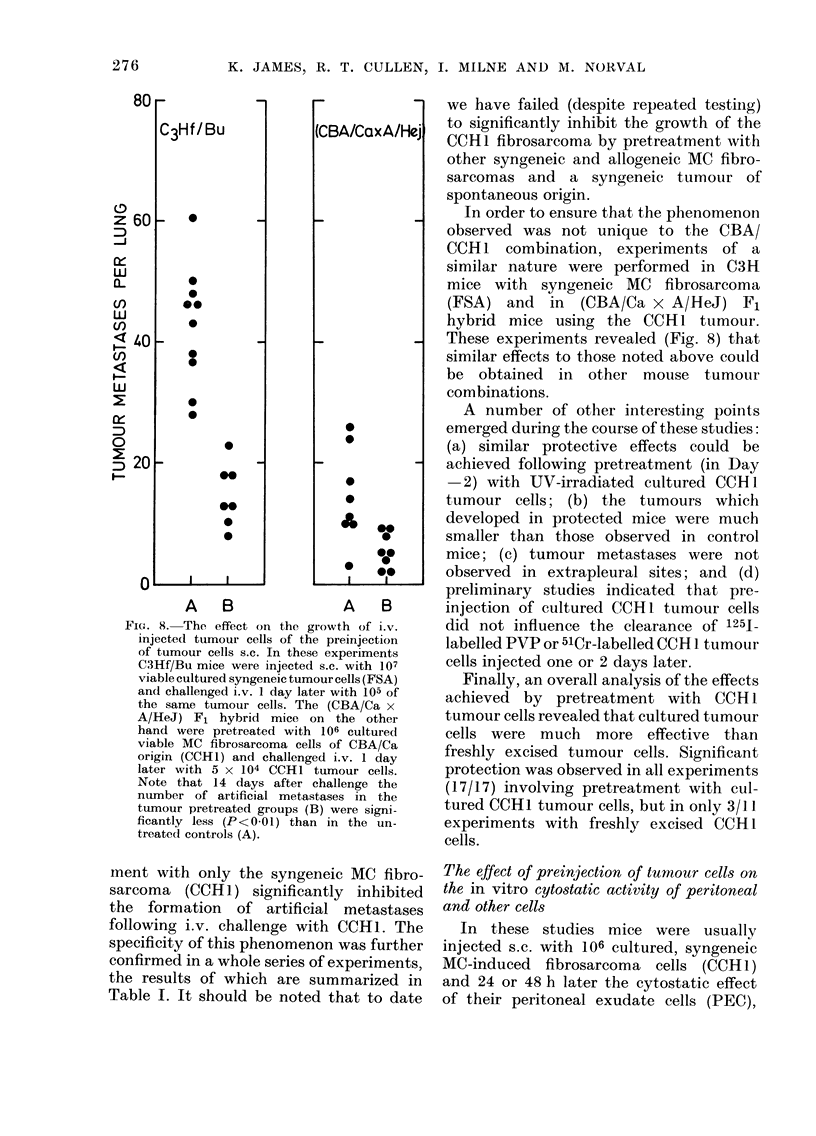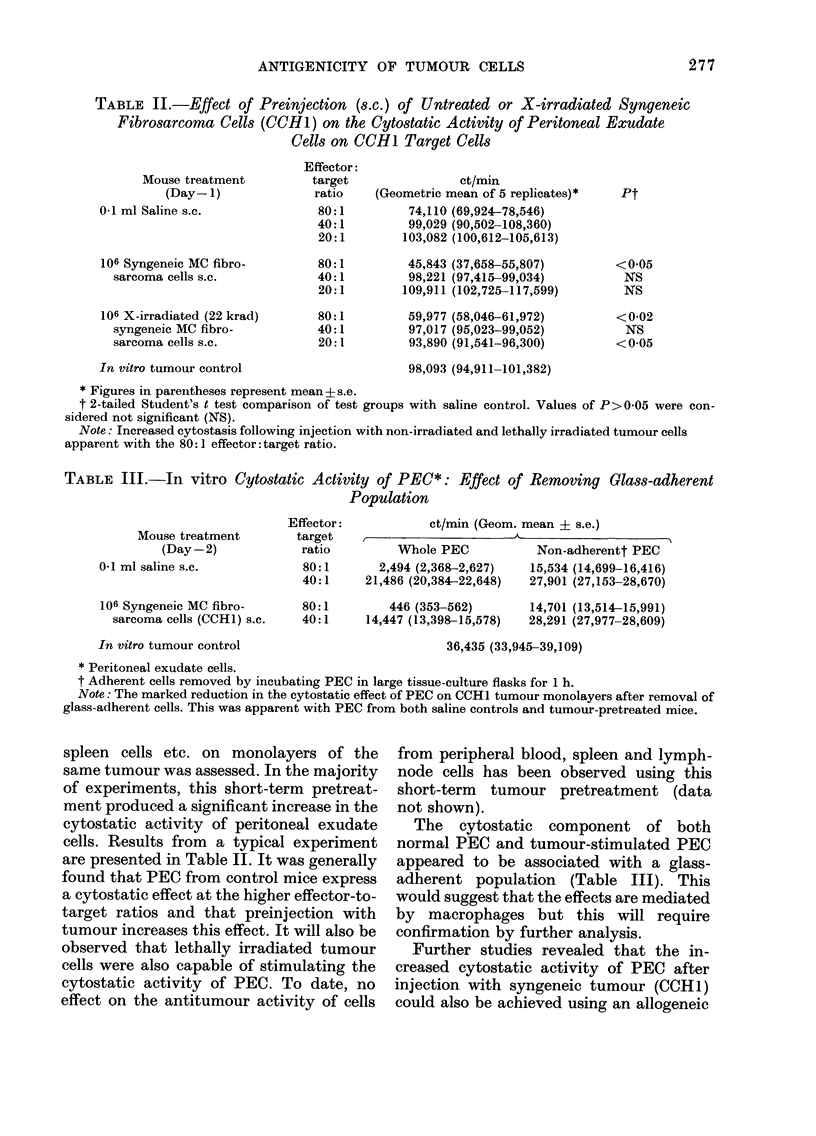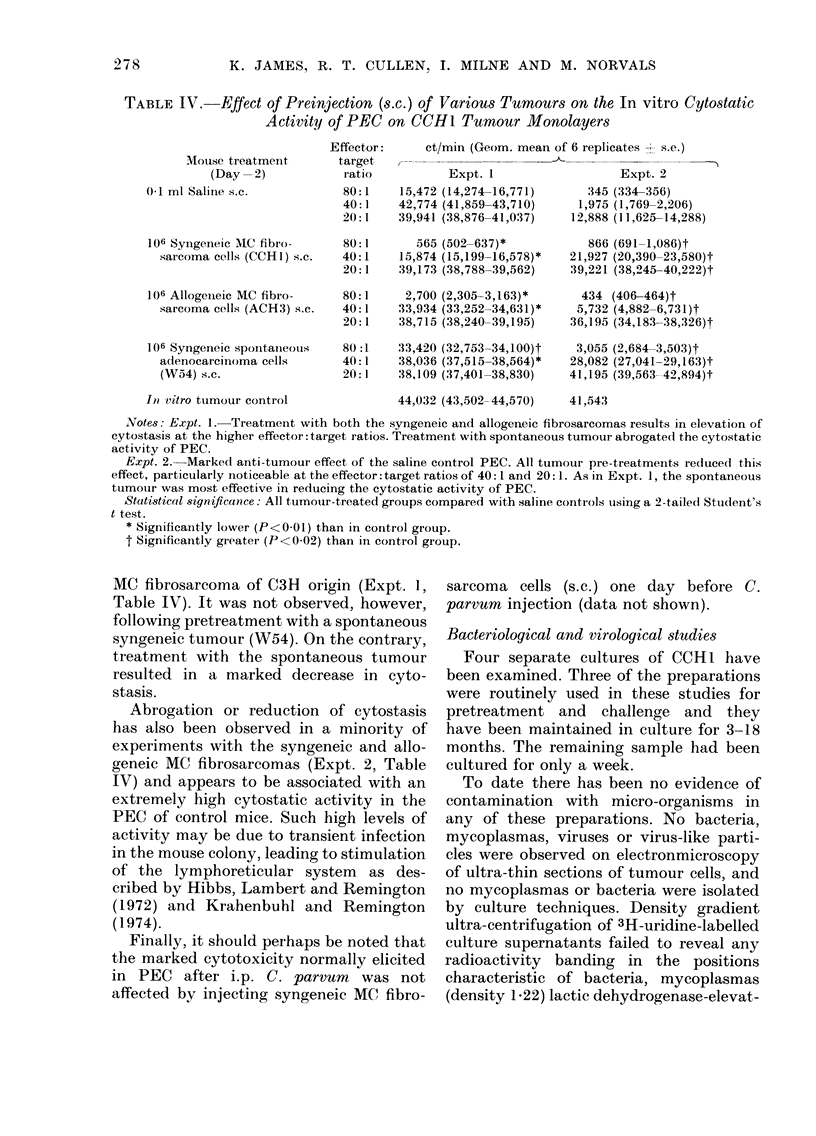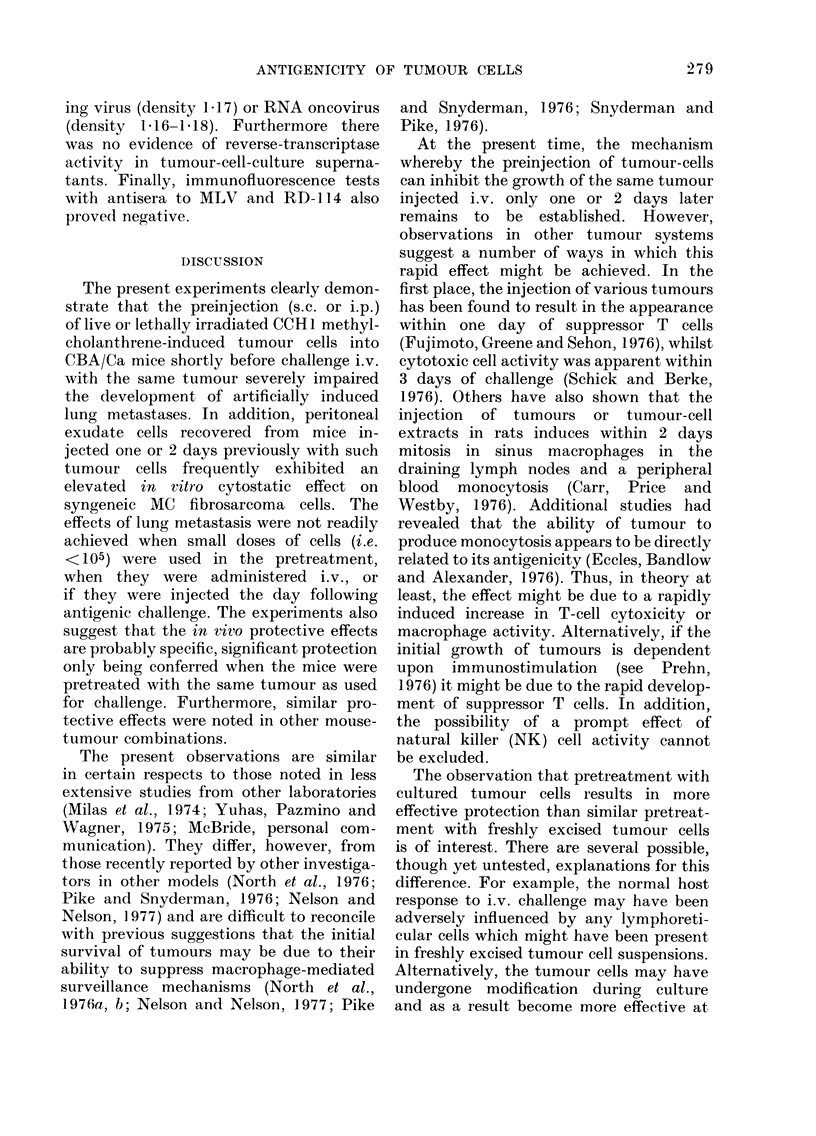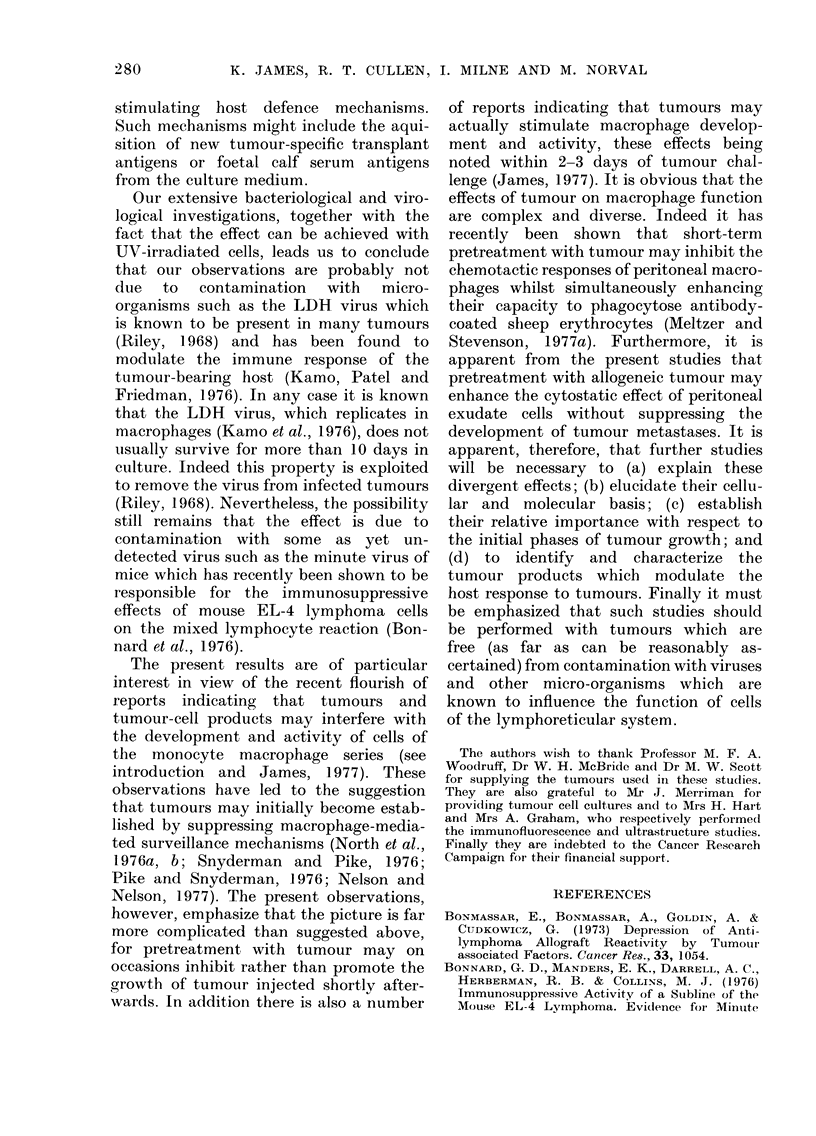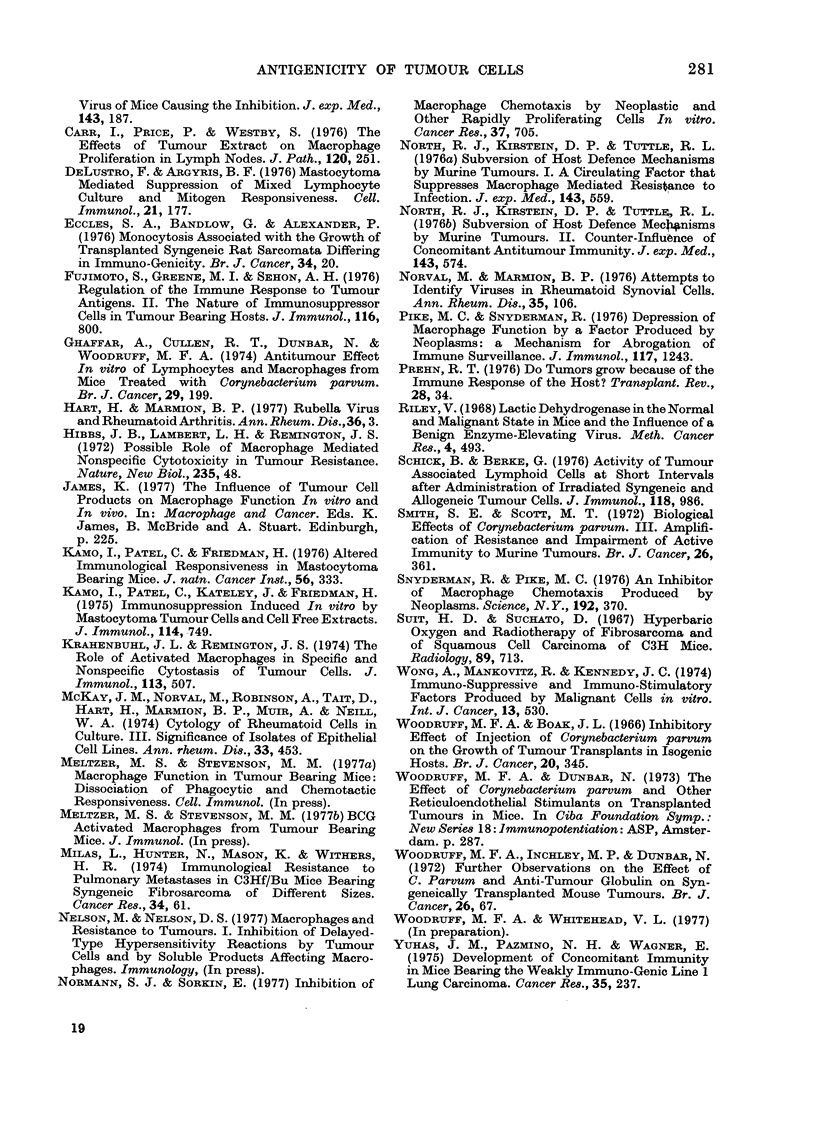# Antitumour responses induced by short-term pretreatment with tumour cells.

**DOI:** 10.1038/bjc.1978.36

**Published:** 1978-02

**Authors:** K. James, R. T. Cullen, I. Milne, M. Norval

## Abstract

The injection (s.c. or i.p.) of 10(6) live or lethally irradiated methylcholanthrene-induced fibrosarcoma cells into CBA/Ca mice one or 2 days before i.v. challenge with the same tumour inhibited the formation of artificial lung tumour metastases. In addition, it also frequently enhanced the cytostatic effect of peritoneal exudate cells on monolayers of the same tumour. The effects on lung tumour metastasis were not noted if X-irradiated tumour was injected i.v., or if s.c. administration was delayed until one day after i.v. challenge. Similar effects on tumour growth were also observed in C3Hf/Bu mice and (CBA/Ca x A/HeJ) F1 hybrids which were pretreated (s.c.) with tumour shortly before i.v. challenge with the same tumour. Further studies in CBA/Ca mice suggested that the protective effect was tumour-specific, for the growth of i.v. injected tumour was not significantly inhibited by pretreatement with a number of other MC-induced or spontaneous tumours from the same and different strains.


Br. J. Cancer (1978) 37, 269

### ANTITUMOUR RESPONSES INDUCED BY SHORT-TERM PRETREATMENT WITH TUMOUR CELLS

K. JAMES, R. T. CULLEN, I. MILNE AND M. NORVAL*

*From the Departments of Surgery and *Bacteriology, University of Edinburgh Medical School, Teviot Place, Edinburgh EH8 9AG, Scotland*

Received 19 September 1977 Accepted 11 October 1977

**Summary.—The injection (s.c. or i.p.) of $10^6$ live or lethally irradiated methylcholanthrene-induced fibrosarcoma cells into CBA/Ca mice one or 2 days before i.v. challenge with the same tumour inhibited the formation of artificial lung tumour metastases. In addition, it also frequently enhanced the cytostatic effect of peritoneal exudate cells on monolayers of the same tumour. The effects on lung tumour metastasis were not 'noted if X-irradiated tumour was injected i.v., or if s.c. administration was delayed until one day after i.v. challenge. Similar effects on tumour growth were also observed in C3Hf/Bu mice and (CBA/Ca × A/HeJ) $F_1$ hybrids which were pretreated (s.c.) with tumour shortly before i.v. challenge with the same tumour. Further studies in CBA/Ca mice suggested that the protective effect was tumour-specific, for the growth of i.v. injected tumour was not significantly inhibited by pretreatment with a number of other MC-induced or spontaneous tumours from the same and different strains.**

A CONSIDERABLE number of investigations undertaken during recent years have shown that factors which suppress the function of lymphoreticular endothelial cell may be released by or extracted from a variety of tumours. For example, factors have been reported which suppress (a) the *in vitro* response of spleen cells to mitogens and alloantigens (De Lustro and Argyris, 1976) and sheep erythrocytes (Wong, Mankovitz and Kennedy, 1974; Kamo *et al.*, 1975); (b) macrophage chemotaxis *in vitro* (Meltzer and Stevenson, 1977*a*, *b*; Normann and Sorkin, 1977; Snydermann and Pike, 1976; Pike and Snydermann, 1976) and *in vivo* (Snyderman and Pike, 1976; Pike and Snyderman, 1976); (c) macrophage-mediated resistance to infection (North, Kirstein and Tuttle, 1976*a*, *b*); and (d) the *in vivo* response to syngeneic (North *et al.*, 1976*b*; Pike and Snyderman, 1976; Nelson and Nelson, 1977) and allogeneic (Bonmassar *et al.*, 1973) tumour transplants. It would also appear that the inhibitory effects observed *in vivo* are not always dependent upon the presence of a large tumour mass, for they may be evident within 24 h of the s.c. transplantation of $10^6$ tumour cells (North *et al.*, 1976*a*, *b*).

The observed effects on macrophage function have led to the suggestion that neoplastic cells may abrogate or interfere with the early phases of immunosurveillance by releasing products which modify the localization and/or activity of macrophages (Snyderman and Pike, 1976; Pike and Snyderman, 1976; North *et al.*, 1976*a*, *b*; Nelson and Nelson, 1977). In view of the above suggestions and the observations of North *et al.* (1976*a*, *b*), we thought it was important to establish whether the injection of syngeneic tumour cells shortly before i.v. challenge with the same tumour cells would increase the incidence of artificial metastases, or interfere with the capacity of macrophages to exert a cytostatic effect on syngeneic target cells *in vitro*. Contrary to expectations we found that the injection of syngeneic tumour cells as late as 1 day before i.v. challenge inhibited the development of artificial metastases. It had, however, a less consistent effect upon the cytostatic activity of peritoneal exudate cells harvested within 2 days of tumour injection.

### MATERIALS AND METHODS

*Mice.*—Almost all the experiments were performed in inbred CBA/Ca mice (male and female) aged 8–12 weeks. The remaining experiments were undertaken either in $F_1$ hybrids of CBA/Ca and A/HeJ mice or inbred C3Hf/Bu mice. The CBA/Ca mice were bred from mice obtained from the MRC Laboratory Animals Centre, Carshalton, Surrey. The A/HeJ mice used in the breeding of the (CBA/Ca × A/HeJ) $F_1$ hybrids were purchased from the Jackson Laboratories, Bar Harbor, Maine, U.S.A. The C3Hf/Bu mice were derived from stock originally obtained from Baylor University, Texas, U.S.A. They were kindly provided by Dr W. H. McBride of the Department of Bacteriology, University of Edinburgh.

*Tumours.*—A number of tumours were used in these studies. They include (a) MC fibrosarcomas from a number of strains of mice; (b) a fibrosarcoma which had been obtained by injecting CBA/Ca mice with syngeneic embryo cells which had transformed *in vitro*; and (c) an adenocarcinoma which had spontaneously appeared in CBA/Ca mice. Further details on the origin, generation number and designation of these tumours are presented in Table I.

Cultured MC-induced CBA fibrosarcoma cells (CCH1) were generally used for pretreatment and always used for challenge. These cells had been maintained for 3–18 months in culture, as previously described (Ghaffar *et al.*, 1974). The T3 fibrosarcoma cell line was also maintained in culture. All the other tumour-cell suspensions used were obtained by pronase digestion of freshly excised tumour cells (Woodruff and Boak, 1966).

*X-Irradiation.*—Tumour cells were irradiated at a dose rate of 274 rad/min to a total of 22,000 rad using a Westinghouse X-ray machine operating at 220 kV and 15 mA, with HVL of 1·2 Cu under conditions of maximum backscatter.

In vivo *experimental model.*—The basic protocol involved injecting mice s.c. with $10^6$ syngeneic or allogeneic tumour cells 1 to 2 days before i.v. challenge with $5 \times 10^4$ tumour cells. The mice were killed 14 days after challenge, the lungs removed and fixed in Bouin's solution and the number of artificial metastases (tumour nodules) per lung counted. Each experimental group contained 6 to 19 mice. Further details on variations in this basic protocol are reported elsewhere in the text or in the footnotes to the figures and tables.

In vitro *cytostatic assay.*—The procedure used has been described in greater detail elsewhere (Ghaffar *et al.*, 1974). In essence it involved the addition of test effector cells to tumour-cell monolayers in the wells of plastic microculture plates and incubating the plates at 37°C for 48 h. At this stage, the culture medium (see below) was replaced by fresh medium containing $^{125}$I-iododeoxyuridine (Radiochemical Centre, Amersham, England). The incorporation of this thymidine analogue into the tumour-cell monolayer was assessed 20 h later in an LKB Wallac gamma scintillometer. The spleen cells whose cytostatic activity was being assessed were obtained by gentle disruption in cold medium in a hand-operated glass homogenizer while the peritoneal exudate cells were obtained by washing out the peritoneal cavity with 3 ml of medium containing 10 u of heparin/ml. These cells were always washed ×3 before use. Throughout these *in vitro* studies, the medium used was RPMI 1640 (Gibco Biocult, Paisley, Scotland) buffered with 20 mM HEPES and supplemented with 10% (vol/vol) foetal calf serum, 100 u/ml penicillin and 100 μg/ml streptomycin.

*Bacteriological and virological investigations.*—A variety of standard procedures were used to ascertain if the cultured CBA MC fibrosarcoma cell line routinely used in these studies (that is, CCH1) was contaminated with micro-organisms which might influence antitumour responses. In brief these included electronmicroscopic examination of tumour cell sections, the screening of cells and culture supernatants for mycoplasma (McKay *et al.*, 1974), reverse transcriptase assays using the method of Dr Natalie Teich (personal communication) and indirect immunofluorescence on acetone-fixed cells (Hart and Marmion, 1977) with antisera to Moloney leukaemia virus and RD-114, an endogenous feline RNA oncovirus. Finally, the cells were grown in the presence of $^3$H-uridine and the supernatants screened for the presence of micro-organisms containing RNA. Further details on the culture procedure and the density-gradient separation technique used in the incorporation studies are reported elsewhere (Norval and Marmion, 1976).

*Presentation of results.*—The number of artificial metastases observed in experimental mice have been presented in scattergram form and the significance of the results has been determined using the Wilcoxon Rank Sum test. The *in vitro* cytostatic results have been expressed as geometric means together with the limits of one standard error from the mean ct/min of 5 replicate samples. The cytostatic indices (CI) have been calculated using the following formula:

$$CI = \frac{(N - T) \times 100}{N}$$

where $N$ represents the mean ct/min obtained using effector cells from untreated (control) mice, and $T$ represents the mean ct/min using effector cells from mice treated with tumour cells. The significance of the cytostatic results has been assessed by the standard Student's two-tail $t$ test.

### RESULTS

### *The effect of the preinjection of tumour cells on the development of artificial lung metastases*

In our initial experiments, mice were injected s.c. with $10^6$ viable cultured tumour cells and challenged i.v. 24 h later with various doses of cultured syngeneic tumour cells. The number of artificially induced pulmonary metastases present 14 days later was determined. The results of one such experiment are presented in Fig. 1. It will be noted that the number of metastases was significantly reduced in mice which had been injected s.c. with syngeneic tumour cells 24 h before i.v. challenge.

These initial observations were unexpected in view of previous suggestions that such pretreatment might interfere with the antitumour activity of host macrophages (for example see North *et al.*, 1976*a*, *b*). We decided therefore to perform additional experiments to see whether the effect observed was dependent upon the dose and route of injection of the tumour cells, the time they were injected in relation to i.v. challenge, and whether or not a similar effect could be achieved with lethally irradiated syngeneic tumour cells or with other syngeneic and allogeneic tumours.

FIG. 1.—The effect on the growth of i.v. injected tumour cells of the preinjection (s.c.) of syngeneic tumour cells. A, C—no pretreatment; B, D—tumour cells s.c. Mice were pretreated by s.c. injection with $10^6$ cultured syngeneic MC fibrosarcoma cells (CCH1) 24 h before i.v. challenge with $5 \times 10^4$ or $1 \times 10^5$ CCH1 tumour cells. Note that 14 days after i.v. challenge the number of lung metastases in the pretreated groups (B and D) was significantly lower ($P < 0{\cdot}01$) than in groups receiving no pretreatment (A and C).

Several experiments were performed to determine the dose dependency of the

FIG. 2.—The effects on the growth of i.v. injected tumour cells of the preinjection (s.c.) of various doses of syngeneic tumour cells. Mice in Groups B, C and D were injected respectively s.c. with $10^7$, $10^6$ and $10^4$ cultured syngeneic MC fibrosarcoma cells (CCH1) 24 h before i.v. challenge with $5 \times 10^4$ CCH1 tumour cells. Group A mice had no pretreatment. Note that 14 days after i.v. challenge the number of metastases in animals pretreated with $10^7$ (B) and $10^6$ (C) tumour cells was significantly lower ($P < 0{\cdot}01$) than in mice receiving no pretreatment (A). The inhibition observed in animals pretreated with $10^4$ tumour cells (D) was just significant at the 0·05 level.

FIG. 3.—The effects on the growth of i.v. injected tumour cells of the preinjection (s.c.) of various tumour cell preparations. All mice received i.v. challenge with $5 \times 10^4$ cultured CCH1 fibrosarcoma cells. Pretreatment: A—no pretreatment; B—syngeneic MC fibrosarcoma cells Day −1; C—lethally irradiated syngeneic MC fibrosarcoma cells Day −1; D—syngeneic MC fibrosarcoma cells Day +1; E—allogeneic MC fibrosarcoma cells Day −1. Syngeneic MC fibrosarcoma cells were from cultured CCH1. Allogeneic MC fibrosarcoma cells were from freshly excised ACH3. Note that 14 days after challenge the number of tumour metastases per lung was significantly lower in mice pretreated with viable or lethally irradiated syngeneic tumour cells (B and C, $P < 0{\cdot}01$). In contrast, pretreatment with allogeneic tumour cells or the administration of tumour cells after i.v. challenge did not inhibit tumour metastases. (For further results of a similar nature see Figs. 5 and 7.)

effect, and the results of one of these experiments are presented in Fig. 2. These studies revealed that $10^6$ tumour cells or more had to be injected s.c. to ensure effective inhibition metastasis.

Experiments were then undertaken to see whether the protection observed above could also be achieved by preinjection of X-irradiated syngeneic tumour cells or allogeneic MC fibrosarcoma cells of A/HeJ origin. These studies revealed that the preinjection of X-irradiated syngeneic tumour cells also inhibited the development of artificial metastases following i.v.

FIG. 4.—The effects on the growth of i.v. injected tumour cells of the preinjection of X-irradiated syngeneic tumour cells by different routes. Mice B–D were injected by the route indicated with $10^6$ lethally irradiated cultured syngeneic MC fibrosarcoma cells (CCH1) and were challenged i.v. 2 days later with $5 \times 10^4$ CCH1 tumour cells. Note that 14 days after challenge the number of lung metastases in the s.c. (B) and i.p. (C) pretreated groups were significantly lower ($P<0 \cdot 01$) than in the control group (A), while those in the i.v. pretreated group (D) were not significantly different ($P<0 \cdot 01$).

FIG. 5.—The effect on the growth of i.v. injected tumour cells of the s.c. injection of syngeneic tumour cells. Mice in Groups B–F were injected s.c. with $10^6$ cultured syngeneic MC fibrosarcoma cells (CCH1) at various times (Day −3 to Day +1 respectively) in relation to challenge i.v. with $5 \times 10^4$ CCH1 tumour cells. Note that the number of tumour metastases 14 days after i.v. challenge was significantly reduced ($P<0 \cdot 01$) in the pretreated groups (B–D) but was not significantly affected in animals injected at the same time (E) or 24 h *after* i.v. challenge (F). See also Fig. 3, (D).

challenge, whilst pretreatment with fibrosarcoma cells of A/HeJ origin (designated ACH3) was without effect. It should also be noted that syngeneic tumour cells failed to inhibit tumour growth if administered after i.v. challenge. This phenomenon was investigated further (see Fig. 5).

Investigations were then made into whether the protective effect afforded by preinjection of syngeneic tumour cells shortly before challenge was dependent upon the route of administration of the original inoculum. In this experiment

FIG. 6.—The effects on the growth of i.v. injected tumour cells of the preinjection (s.c.) of syngeneic tumour cells. Mice in Groups B–D were injected s.c. with $10^6$ cultured syngeneic MC fibrosarcoma cells (CCH1) at various times (Days −14, −7 and −2 respectively) in relation to i.v. challenge with $5 \times 10^4$ CCH1 tumour cells. Note that the number of tumour metastases observed 14 days after i.v. challenge was significantly decreased in all pretreated groups ($P<0{\cdot}05$ to $P<0{\cdot}01$).

mice were injected s.c., i.p. or i.v. with $10^6$ irradiated tumour cells. Significant inhibition of pulmonary metastasis was noted following preinjection by the s.c. and i.p. routes, but not by the i.v. route (see Fig. 4). This effect has been observed on 3 separate occasions.

The next 2 experiments were prompted by previous observations that the pre-

FIG. 7.—The effect on the growth of i.v. injected MC fibrosarcoma cells of the preinjection (s.c.) of various tumour cells. Mice were injected s.c. with $10^6$ viable cells from a variety of freshly excised tumours and challenged i.v. 48 h later with $5 \times 10^4$ cultured MC fibrosarcoma cells (CCH1). Pretreatment: A—no pretreatment: B—syngeneic MC fibrosarcoma (CCH1); C—syngeneic spontaneous tumour (W54); D—allogeneic MC fibrosarcoma (ACH3); E—allogeneic MC fibrosarcoma (FSA). Note that 14 days after challenge the only significant reduction in lung metastasis was observed in mice pretreated with CCH1 tumour (B, $P<0{\cdot}01$). On this occasion the spontaneous tumour (W54, C) also inhibited tumour growth, though the effect was not significant, and in the 7 additional experiments performed with this tumour the inhibitory effects were much less marked.

TABLE I.—*A Summary of the Effects of Preinjection* (s.c.) of Various Tumours on the Growth in CBA Mice of a Syngeneic MC Fibrosarcoma (CCH1) Injected (i.v.) 24–48 h Later*

| The tumours preinjected | | | | | |
|---|---|---|---|---|---|
| Description | Tumour designation | Generation Nos. | Strain of origin | How induced | Times inhibited/ times tested† |
| Fibrosarcoma | CCH1 | 18–19 | CBA/Ca | With 3 methylcholanthrene‡ (Woodruff, Inchley and Dunbar, 1972) | 20§/28 |
| Fibrosarcoma | CCH5 | 1–2 | CBA/Ca | With 3 methylcholanthrene (Woodruff *et al.*, 1972) | 0/3 |
| Fibrosarcoma | T3 | ¶ | CBA/p | By injection of embryo cells spontaneously transformed *in vitro* (Smith and Scott, 1972) | 0/1 |
| Adenocarcinoma | W54 | 4–5 | CBA/Ca | Spontaneous origin (Woodruff and Whitehead, 1977) (in preparation) | 0/8 |
| Fibrosarcoma | ACH3 | 26–27 | A/HeJ | With 3 methylcholanthrene (Woodruff *et al.*, 1972) | 0/8 |
| Fibrosarcoma | FSA | 6 | C3Hf/Bu | With 3 methylcholanthrene (Suit and Suchato, 1967) | 0/3 |

* Mice were injected s.c. with $10^6$ viable tumour cells and 24–48 h later challenged i.v. with $5 \times 10^4$ viable CCH1 fibrosarcoma cells.

† The number of experiments in which significant inhibition ($P < 0{\cdot}05$) of lung metastases formation was observed out of the total number of experiments performed.

‡ This tumour is highly immunogenic, a single inoculum of $10^6$ irradiated cells (22,000 rad) conferring complete protection to mice challenged 2 weeks later with $10^4$ viable tumour cells (Woodruff and Dunbar, 1973).

§ In all 28 experiments the growth of tumour was inhibited, but this inhibition failed to reach significant levels in 8 experiments. On all these occasions a suspension of freshly excised tumour cells had been injected.

¶ Maintained in culture.

*Note:* Significant inhibition of growth of i.v.-injected cultured CCH1 tumour cells is only achieved after preinjection of CCH1 tumour cells.

ence of s.c. growing tumour reduced the number of artificial pulmonary metastases which could be induced by the i.v. injection of syngeneic tumour cells (Milas *et al.*, 1974). These workers also demonstrated by adoptive transfer that the resistance conferred was immunologically mediated, protection being most effectively transferred by cells from mice challenged 12 days previously with viable tumour cells. We decided therefore to investigate in more detail the development of artificial pulmonary metastases in mice which had been injected s.c. with syngeneic tumour cells at various times before and after i.v. challenge.

The initial "short-term" pretreatment experiment involved injecting mice s.c. with $10^6$ viable syngeneic tumour cells on either Day −3, −2, −1, 0 or +1 in relation to i.v. challenge with $5 \times 10^4$ tumour cells, and 2 weeks after challenge the number of lung metastases formed was assessed. Significant suppression of metastasis was observed in all mice pretreated with syngeneic tumour cells, but was not achieved in mice treated after i.v. challenge (Fig. 5), thus confirming our preliminary observation (Fig. 3).

A further experiment was performed in which mice were injected s.c. with $10^6$ viable syngeneic tumour cells 14, 7 and 2 days before i.v. challenge. A significant reduction in pulmonary metastasis was noted following pretreatment at all these times.

The specificity of the protection conferred was assessed by pretreating the CBA mice with a variety of tumours of syngeneic and allogeneic origin. The results of one such experiment are shown in Fig. 7. It will be observed that pretreat-

FIG. 8.—The effect on the growth of i.v. injected tumour cells of the preinjection of tumour cells s.c. In these experiments C3Hf/Bu mice were injected s.c. with $10^7$ viable cultured syngeneic tumour cells (FSA) and challenged i.v. 1 day later with $10^5$ of the same tumour cells. The (CBA/Ca $\times$ A/HeJ) $F_1$ hybrid mice on the other hand were pretreated with $10^6$ cultured viable MC fibrosarcoma cells of CBA/Ca origin (CCH1) and challenged i.v. 1 day later with $5 \times 10^4$ CCH1 tumour cells. Note that 14 days after challenge the number of artificial metastases in the tumour pretreated groups (B) were significantly less ($P<0{\cdot}01$) than in the untreated controls (A).

ment with only the syngeneic MC fibrosarcoma (CCH1) significantly inhibited the formation of artificial metastases following i.v. challenge with CCH1. The specificity of this phenomenon was further confirmed in a whole series of experiments, the results of which are summarized in Table I. It should be noted that to date we have failed (despite repeated testing) to significantly inhibit the growth of the CCH1 fibrosarcoma by pretreatment with other syngeneic and allogeneic MC fibrosarcomas and a syngeneic tumour of spontaneous origin.

In order to ensure that the phenomenon observed was not unique to the CBA/CCH1 combination, experiments of a similar nature were performed in C3H mice with syngeneic MC fibrosarcoma (FSA) and in (CBA/Ca $\times$ A/HeJ) $F_1$ hybrid mice using the CCH1 tumour. These experiments revealed (Fig. 8) that similar effects to those noted above could be obtained in other mouse tumour combinations.

A number of other interesting points emerged during the course of these studies: (a) similar protective effects could be achieved following pretreatment (in Day −2) with UV-irradiated cultured CCH1 tumour cells; (b) the tumours which developed in protected mice were much smaller than those observed in control mice; (c) tumour metastases were not observed in extrapleural sites; and (d) preliminary studies indicated that preinjection of cultured CCH1 tumour cells did not influence the clearance of $^{125}$I-labelled PVP or $^{51}$Cr-labelled CCH1 tumour cells injected one or 2 days later.

Finally, an overall analysis of the effects achieved by pretreatment with CCH1 tumour cells revealed that cultured tumour cells were much more effective than freshly excised tumour cells. Significant protection was observed in all experiments (17/17) involving pretreatment with cultured CCH1 tumour cells, but in only 3/11 experiments with freshly excised CCH1 cells.

*The effect of preinjection of tumour cells on the* in vitro *cytostatic activity of peritoneal and other cells*

In these studies mice were usually injected s.c. with $10^6$ cultured, syngeneic MC-induced fibrosarcoma cells (CCH1) and 24 or 48 h later the cytostatic effect of their peritoneal exudate cells (PEC),

TABLE II.—*Effect of Preinjection (s.c.) of Untreated or X-irradiated Syngeneic Fibrosarcoma Cells (CCH1) on the Cytostatic Activity of Peritoneal Exudate Cells on CCH1 Target Cells*

| Mouse treatment (Day − 1) | Effector: target ratio | ct/min (Geometric mean of 5 replicates)* | $P$† |
|---|---|---|---|
| 0·1 ml Saline s.c. | 80:1 | 74,110 (69,924–78,546) | |
| | 40:1 | 99,029 (90,502–108,360) | |
| | 20:1 | 103,082 (100,612–105,613) | |
| $10^6$ Syngeneic MC fibrosarcoma cells s.c. | 80:1 | 45,843 (37,658–55,807) | <0·05 |
| | 40:1 | 98,221 (97,415–99,034) | NS |
| | 20:1 | 109,911 (102,725–117,599) | NS |
| $10^6$ X-irradiated (22 krad) syngeneic MC fibrosarcoma cells s.c. | 80:1 | 59,977 (58,046–61,972) | <0·02 |
| | 40:1 | 97,017 (95,023–99,052) | NS |
| | 20:1 | 93,890 (91,541–96,300) | <0·05 |
| *In vitro* tumour control | | 98,093 (94,911–101,382) | |

* Figures in parentheses represent mean ± s.e.

† 2-tailed Student's $t$ test comparison of test groups with saline control. Values of $P > 0{\cdot}05$ were considered not significant (NS).

*Note:* Increased cytostasis following injection with non-irradiated and lethally irradiated tumour cells apparent with the 80:1 effector:target ratio.

TABLE III.—In vitro *Cytostatic Activity of PEC*: Effect of Removing Glass-adherent Population*

| Mouse treatment (Day − 2) | Effector: target ratio | ct/min (Geom. mean ± s.e.) Whole PEC | Non-adherent† PEC |
|---|---|---|---|
| 0·1 ml saline s.c. | 80:1 | 2,494 (2,368–2,627) | 15,534 (14,699–16,416) |
| | 40:1 | 21,486 (20,384–22,648) | 27,901 (27,153–28,670) |
| $10^6$ Syngeneic MC fibrosarcoma cells (CCH1) s.c. | 80:1 | 446 (353–562) | 14,701 (13,514–15,991) |
| | 40:1 | 14,447 (13,398–15,578) | 28,291 (27,977–28,609) |
| *In vitro* tumour control | | 36,435 (33,945–39,109) | |

* Peritoneal exudate cells.

† Adherent cells removed by incubating PEC in large tissue-culture flasks for 1 h.

*Note:* The marked reduction in the cytostatic effect of PEC on CCH1 tumour monolayers after removal of glass-adherent cells. This was apparent with PEC from both saline controls and tumour-pretreated mice.

spleen cells etc. on monolayers of the same tumour was assessed. In the majority of experiments, this short-term pretreatment produced a significant increase in the cytostatic activity of peritoneal exudate cells. Results from a typical experiment are presented in Table II. It was generally found that PEC from control mice express a cytostatic effect at the higher effector-to-target ratios and that preinjection with tumour increases this effect. It will also be observed that lethally irradiated tumour cells were also capable of stimulating the cytostatic activity of PEC. To date, no effect on the antitumour activity of cells from peripheral blood, spleen and lymph-node cells has been observed using this short-term tumour pretreatment (data not shown).

The cytostatic component of both normal PEC and tumour-stimulated PEC appeared to be associated with a glass-adherent population (Table III). This would suggest that the effects are mediated by macrophages but this will require confirmation by further analysis.

Further studies revealed that the increased cytostatic activity of PEC after injection with syngeneic tumour (CCH1) could also be achieved using an allogeneic

TABLE IV.—*Effect of Preinjection (s.c.) of Various Tumours on the* In vitro *Cytostatic Activity of PEC on CCH*1 *Tumour Monolayers*

| Mouse treatment (Day −2) | Effector: target ratio | ct/min (Geom. mean of 6 replicates ± s.e.) Expt. 1 | Expt. 2 |
|---|---|---|---|
| 0·1 ml Saline s.c. | 80:1 | 15,472 (14,274–16,771) | 345 (334–356) |
| | 40:1 | 42,774 (41,859–43,710) | 1,975 (1,769–2,206) |
| | 20:1 | 39,941 (38,876–41,037) | 12,888 (11,625–14,288) |
| $10^6$ Syngeneic MC fibrosarcoma cells (CCH1) s.c. | 80:1 | 565 (502–637)* | 866 (691–1,086)† |
| | 40:1 | 15,874 (15,199–16,578)* | 21,927 (20,390–23,580)† |
| | 20:1 | 39,173 (38,788–39,562) | 39,221 (38,245–40,222)† |
| $10^6$ Allogeneic MC fibrosarcoma cells (ACH3) s.c. | 80:1 | 2,700 (2,305–3,163)* | 434 (406–464)† |
| | 40:1 | 33,934 (33,252–34,631)* | 5,732 (4,882–6,731)† |
| | 20:1 | 38,715 (38,240–39,195) | 36,195 (34,183–38,326)† |
| $10^6$ Syngeneic spontaneous adenocarcinoma cells (W54) s.c. | 80:1 | 33,420 (32,753–34,100)† | 3,055 (2,684–3,503)† |
| | 40:1 | 38,036 (37,515–38,564)* | 28,082 (27,041–29,163)† |
| | 20:1 | 38,109 (37,401–38,830) | 41,195 (39,563–42,894)† |
| *In vitro* tumour control | | 44,032 (43,502–44,570) | 41,543 |

*Notes: Expt.* 1.—Treatment with both the syngeneic and allogeneic fibrosarcomas results in elevation of cytostasis at the higher effector:target ratios. Treatment with spontaneous tumour abrogated the cytostatic activity of PEC.

*Expt.* 2.—Marked anti-tumour effect of the saline control PEC. All tumour pre-treatments reduced this effect, particularly noticeable at the effector:target ratios of 40:1 and 20:1. As in Expt. 1, the spontaneous tumour was most effective in reducing the cytostatic activity of PEC.

*Statistical significance:* All tumour-treated groups compared with saline controls using a 2-tailed Student's *t* test.

* Significantly lower ($P<0·01$) than in control group.

† Significantly greater ($P<0·02$) than in control group.

MC fibrosarcoma of C3H origin (Expt. 1, Table IV). It was not observed, however, following pretreatment with a spontaneous syngeneic tumour (W54). On the contrary, treatment with the spontaneous tumour resulted in a marked decrease in cytostasis.

Abrogation or reduction of cytostasis has also been observed in a minority of experiments with the syngeneic and allogeneic MC fibrosarcomas (Expt. 2, Table IV) and appears to be associated with an extremely high cytostatic activity in the PEC of control mice. Such high levels of activity may be due to transient infection in the mouse colony, leading to stimulation of the lymphoreticular system as described by Hibbs, Lambert and Remington (1972) and Krahenbuhl and Remington (1974).

Finally, it should perhaps be noted that the marked cytotoxicity normally elicited in PEC after i.p. *C. parvum* was not affected by injecting syngeneic MC fibrosarcoma cells (s.c.) one day before *C. parvum* injection (data not shown).

*Bacteriological and virological studies*

Four separate cultures of CCH1 have been examined. Three of the preparations were routinely used in these studies for pretreatment and challenge and they have been maintained in culture for 3–18 months. The remaining sample had been cultured for only a week.

To date there has been no evidence of contamination with micro-organisms in any of these preparations. No bacteria, mycoplasmas, viruses or virus-like particles were observed on electronmicroscopy of ultra-thin sections of tumour cells, and no mycoplasmas or bacteria were isolated by culture techniques. Density gradient ultra-centrifugation of $^3$H-uridine-labelled culture supernatants failed to reveal any radioactivity banding in the positions characteristic of bacteria, mycoplasmas (density 1·22) lactic dehydrogenase-elevating virus (density 1·17) or RNA oncovirus (density 1·16–1·18). Furthermore there was no evidence of reverse-transcriptase activity in tumour-cell-culture supernatants. Finally, immunofluorescence tests with antisera to MLV and RD-114 also proved negative.

### DISCUSSION

The present experiments clearly demonstrate that the preinjection (s.c. or i.p.) of live or lethally irradiated CCH1 methylcholanthrene-induced tumour cells into CBA/Ca mice shortly before challenge i.v. with the same tumour severely impaired the development of artificially induced lung metastases. In addition, peritoneal exudate cells recovered from mice injected one or 2 days previously with such tumour cells frequently exhibited an elevated *in vitro* cytostatic effect on syngeneic MC fibrosarcoma cells. The effects of lung metastasis were not readily achieved when small doses of cells (*i.e.* $<10^5$) were used in the pretreatment, when they were administered i.v., or if they were injected the day following antigenic challenge. The experiments also suggest that the *in vivo* protective effects are probably specific, significant protection only being conferred when the mice were pretreated with the same tumour as used for challenge. Furthermore, similar protective effects were noted in other mouse-tumour combinations.

The present observations are similar in certain respects to those noted in less extensive studies from other laboratories (Milas *et al.*, 1974; Yuhas, Pazmino and Wagner, 1975; McBride, personal communication). They differ, however, from those recently reported by other investigators in other models (North *et al.*, 1976; Pike and Snyderman, 1976; Nelson and Nelson, 1977) and are difficult to reconcile with previous suggestions that the initial survival of tumours may be due to their ability to suppress macrophage-mediated surveillance mechanisms (North *et al.*, 1976*a*, *b*; Nelson and Nelson, 1977; Pike and Snyderman, 1976; Snyderman and Pike, 1976).

At the present time, the mechanism whereby the preinjection of tumour-cells can inhibit the growth of the same tumour injected i.v. only one or 2 days later remains to be established. However, observations in other tumour systems suggest a number of ways in which this rapid effect might be achieved. In the first place, the injection of various tumours has been found to result in the appearance within one day of suppressor T cells (Fujimoto, Greene and Sehon, 1976), whilst cytotoxic cell activity was apparent within 3 days of challenge (Schick and Berke, 1976). Others have also shown that the injection of tumours or tumour-cell extracts in rats induces within 2 days mitosis in sinus macrophages in the draining lymph nodes and a peripheral blood monocytosis (Carr, Price and Westby, 1976). Additional studies had revealed that the ability of tumour to produce monocytosis appears to be directly related to its antigenicity (Eccles, Bandlow and Alexander, 1976). Thus, in theory at least, the effect might be due to a rapidly induced increase in T-cell cytoxicity or macrophage activity. Alternatively, if the initial growth of tumours is dependent upon immunostimulation (see Prehn, 1976) it might be due to the rapid development of suppressor T cells. In addition, the possibility of a prompt effect of natural killer (NK) cell activity cannot be excluded.

The observation that pretreatment with cultured tumour cells results in more effective protection than similar pretreatment with freshly excised tumour cells is of interest. There are several possible, though yet untested, explanations for this difference. For example, the normal host response to i.v. challenge may have been adversely influenced by any lymphoreticular cells which might have been present in freshly excised tumour cell suspensions. Alternatively, the tumour cells may have undergone modification during culture and as a result become more effective at stimulating host defence mechanisms. Such mechanisms might include the aquisition of new tumour-specific transplant antigens or foetal calf serum antigens from the culture medium.

Our extensive bacteriological and virological investigations, together with the fact that the effect can be achieved with UV-irradiated cells, leads us to conclude that our observations are probably not due to contamination with micro-organisms such as the LDH virus which is known to be present in many tumours (Riley, 1968) and has been found to modulate the immune response of the tumour-bearing host (Kamo, Patel and Friedman, 1976). In any case it is known that the LDH virus, which replicates in macrophages (Kamo *et al.*, 1976), does not usually survive for more than 10 days in culture. Indeed this property is exploited to remove the virus from infected tumours (Riley, 1968). Nevertheless, the possibility still remains that the effect is due to contamination with some as yet undetected virus such as the minute virus of mice which has recently been shown to be responsible for the immunosuppressive effects of mouse EL-4 lymphoma cells on the mixed lymphocyte reaction (Bonnard *et al.*, 1976).

The present results are of particular interest in view of the recent flourish of reports indicating that tumours and tumour-cell products may interfere with the development and activity of cells of the monocyte macrophage series (see introduction and James, 1977). These observations have led to the suggestion that tumours may initially become established by suppressing macrophage-mediated surveillance mechanisms (North *et al.*, 1976*a*, *b*; Snyderman and Pike, 1976; Pike and Snyderman, 1976; Nelson and Nelson, 1977). The present observations, however, emphasize that the picture is far more complicated than suggested above, for pretreatment with tumour may on occasions inhibit rather than promote the growth of tumour injected shortly afterwards. In addition there is also a number of reports indicating that tumours may actually stimulate macrophage development and activity, these effects being noted within 2–3 days of tumour challenge (James, 1977). It is obvious that the effects of tumour on macrophage function are complex and diverse. Indeed it has recently been shown that short-term pretreatment with tumour may inhibit the chemotactic responses of peritoneal macrophages whilst simultaneously enhancing their capacity to phagocytose antibody-coated sheep erythrocytes (Meltzer and Stevenson, 1977*a*). Furthermore, it is apparent from the present studies that pretreatment with allogeneic tumour may enhance the cytostatic effect of peritoneal exudate cells without suppressing the development of tumour metastases. It is apparent, therefore, that further studies will be necessary to (a) explain these divergent effects; (b) elucidate their cellular and molecular basis; (c) establish their relative importance with respect to the initial phases of tumour growth; and (d) to identify and characterize the tumour products which modulate the host response to tumours. Finally it must be emphasized that such studies should be performed with tumours which are free (as far as can be reasonably ascertained) from contamination with viruses and other micro-organisms which are known to influence the function of cells of the lymphoreticular system.

The authors wish to thank Professor M. F. A. Woodruff, Dr W. H. McBride and Dr M. W. Scott for supplying the tumours used in these studies. They are also grateful to Mr J. Merriman for providing tumour cell cultures and to Mrs H. Hart and Mrs A. Graham, who respectively performed the immunofluorescence and ultrastructure studies. Finally they are indebted to the Cancer Research Campaign for their financial support.

### REFERENCES

Bonmassar, E., Bonmassar, A., Goldin, A. & Cudkowicz, G. (1973) Depression of Anti-lymphoma Allograft Reactivity by Tumour associated Factors. *Cancer Res.*, **33**, 1054.

Bonnard, G. D., Manders, E. K., Darrell, A. C., Herberman, R. B. & Collins, M. J. (1976) Immunosuppressive Activity of a Subline of the Mouse EL-4 Lymphoma. Evidence for Minute

Virus of Mice Causing the Inhibition. *J. exp. Med.*, **143**, 187.
CARR, I., PRICE, P. & WESTBY, S. (1976) The Effects of Tumour Extract on Macrophage Proliferation in Lymph Nodes. *J. Path.*, **120**, 251.
DELUSTRO, F. & ARGYRIS, B. F. (1976) Mastocytoma Mediated Suppression of Mixed Lymphocyte Culture and Mitogen Responsiveness. *Cell. Immunol.*, **21**, 177.
ECCLES, S. A., BANDLOW, G. & ALEXANDER, P. (1976) Monocytosis Associated with the Growth of Transplanted Syngeneic Rat Sarcomata Differing in Immuno-Genicity. *Br. J. Cancer*, **34**, 20.
FUJIMOTO, S., GREENE, M. I. & SEHON, A. H. (1976) Regulation of the Immune Response to Tumour Antigens. II. The Nature of Immunosuppressor Cells in Tumour Bearing Hosts. *J. Immunol.*, **116**, 800.
GHAFFAR, A., CULLEN, R. T., DUNBAR, N. & WOODRUFF, M. F. A. (1974) Antitumour Effect *In vitro* of Lymphocytes and Macrophages from Mice Treated with *Corynebacterium parvum*. *Br. J. Cancer*, **29**, 199.
HART, H. & MARMION, B. P. (1977) Rubella Virus and Rheumatoid Arthritis. *Ann. Rheum. Dis.*, **36**, 3.
HIBBS, J. B., LAMBERT, L. H. & REMINGTON, J. S. (1972) Possible Role of Macrophage Mediated Nonspecific Cytotoxicity in Tumour Resistance. *Nature, New Biol.*, **235**, 48.
JAMES, K. (1977) The Influence of Tumour Cell Products on Macrophage Function *In vitro* and *In vivo*. In: *Macrophage and Cancer*. Eds. K. James, B. McBride and A. Stuart. Edinburgh, p. 225.
KAMO, I., PATEL, C. & FRIEDMAN, H. (1976) Altered Immunological Responsiveness in Mastocytoma Bearing Mice. *J. natn. Cancer Inst.*, **56**, 333.
KAMO, I., PATEL, C., KATELEY, J. & FRIEDMAN, H. (1975) Immunosuppression Induced *In vitro* by Mastocytoma Tumour Cells and Cell Free Extracts. *J. Immunol.*, **114**, 749.
KRAHENBUHL, J. L. & REMINGTON, J. S. (1974) The Role of Activated Macrophages in Specific and Nonspecific Cytostasis of Tumour Cells. *J. Immunol.*, **113**, 507.
MCKAY, J. M., NORVAL, M., ROBINSON, A., TAIT, D., HART, H., MARMION, B. P., MUIR, A. & NEILL, W. A. (1974) Cytology of Rheumatoid Cells in Culture. III. Significance of Isolates of Epithelial Cell Lines. *Ann. rheum. Dis.*, **33**, 453.
MELTZER, M. S. & STEVENSON, M. M. (1977*a*) Macrophage Function in Tumour Bearing Mice: Dissociation of Phagocytic and Chemotactic Responsiveness. *Cell. Immunol.* (In press).
MELTZER, M. S. & STEVENSON, M. M. (1977*b*) BCG Activated Macrophages from Tumour Bearing Mice. *J. Immunol.* (In press).
MILAS, L., HUNTER, N., MASON, K. & WITHERS, H. R. (1974) Immunological Resistance to Pulmonary Metastases in C3Hf/Bu Mice Bearing Syngeneic Fibrosarcoma of Different Sizes. *Cancer Res.*, **34**, 61.
NELSON, M. & NELSON, D. S. (1977) Macrophages and Resistance to Tumours. I. Inhibition of Delayed-Type Hypersensitivity Reactions by Tumour Cells and by Soluble Products Affecting Macrophages. *Immunology*, (In press).
NORMANN, S. J. & SORKIN, E. (1977) Inhibition of Macrophage Chemotaxis by Neoplastic and Other Rapidly Proliferating Cells *In vitro*. *Cancer Res.*, **37**, 705.
NORTH, R. J., KIRSTEIN, D. P. & TUTTLE, R. L. (1976*a*) Subversion of Host Defence Mechanisms by Murine Tumours. I. A Circulating Factor that Suppresses Macrophage Mediated Resistance to Infection. *J. exp. Med.*, **143**, 559.
NORTH, R. J., KIRSTEIN, D. P. & TUTTLE, R. L. (1976*b*) Subversion of Host Defence Mechanisms by Murine Tumours. II. Counter-Influence of Concomitant Antitumour Immunity. *J. exp. Med.*, **143**, 574.
NORVAL, M. & MARMION, B. P. (1976) Attempts to Identify Viruses in Rheumatoid Synovial Cells. *Ann. Rheum. Dis.*, **35**, 106.
PIKE, M. C. & SNYDERMAN, R. (1976) Depression of Macrophage Function by a Factor Produced by Neoplasms: a Mechanism for Abrogation of Immune Surveillance. *J. Immunol.*, **117**, 1243.
PREHN, R. T. (1976) Do Tumors grow because of the Immune Response of the Host? *Transplant. Rev.*, **28**, 34.
RILEY, V. (1968) Lactic Dehydrogenase in the Normal and Malignant State in Mice and the Influence of a Benign Enzyme-Elevating Virus. *Meth. Cancer Res.*, **4**, 493.
SCHICK, B. & BERKE, G. (1976) Activity of Tumour Associated Lymphoid Cells at Short Intervals after Administration of Irradiated Syngeneic and Allogeneic Tumour Cells. *J. Immunol.*, **118**, 986.
SMITH, S. E. & SCOTT, M. T. (1972) Biological Effects of *Corynebacterium parvum*. III. Amplification of Resistance and Impairment of Active Immunity to Murine Tumours. *Br. J. Cancer*, **26**, 361.
SNYDERMAN, R. & PIKE, M. C. (1976) An Inhibitor of Macrophage Chemotaxis Produced by Neoplasms. *Science, N.Y.*, **192**, 370.
SUIT, H. D. & SUCHATO, D. (1967) Hyperbaric Oxygen and Radiotherapy of Fibrosarcoma and of Squamous Cell Carcinoma of C3H Mice. *Radiology*, **89**, 713.
WONG, A., MANKOVITZ, R. & KENNEDY, J. C. (1974) Immuno-Suppressive and Immuno-Stimulatory Factors Produced by Malignant Cells *in vitro*. *Int. J. Cancer*, **13**, 530.
WOODRUFF, M. F. A. & BOAK, J. L. (1966) Inhibitory Effect of Injection of *Corynebacterium parvum* on the Growth of Tumour Transplants in Isogenic Hosts. *Br. J. Cancer*, **20**, 345.
WOODRUFF, M. F. A. & DUNBAR, N. (1973) The Effect of *Corynebacterium parvum* and Other Reticuloendothelial Stimulants on Transplanted Tumours in Mice. In *Ciba Foundation Symp.: New Series* 18: *Immunopotentiation*: ASP, Amsterdam. p. 287.
WOODRUFF, M. F. A., INCHLEY, M. P. & DUNBAR, N. (1972) Further Observations on the Effect of *C. Parvum* and Anti-Tumour Globulin on Syngeneically Transplanted Mouse Tumours. *Br. J. Cancer*, **26**, 67.
WOODRUFF, M. F. A. & WHITEHEAD, V. L. (1977) (In preparation).
YUHAS, J. M., PAZMINO, N. H. & WAGNER, E. (1975) Development of Concomitant Immunity in Mice Bearing the Weakly Immuno-Genic Line 1 Lung Carcinoma. *Cancer Res.*, **35**, 237.